# Recurrent Pneumothorax in a Post-pneumonectomy Patient: Surgical Management Under Extracorporeal Membrane Oxygenation Support

**DOI:** 10.7759/cureus.100814

**Published:** 2026-01-05

**Authors:** Anita Paiva, Maria Jacob, Mariana G Ribeiro, Roberto Roncon-Albuquerque, Pedro Fernandes

**Affiliations:** 1 Cardiothoracic Surgery, Unidade Local de Saúde de São João, Porto, PRT; 2 Pulmonology, Unidade Local de Saúde de São João, Porto, PRT; 3 Intensive Care Unit, Unidade Local de Saúde de São João, Porto, PRT

**Keywords:** contralateral pneumothorax post-pneumonectomy, prolonged air leak, thoracic surgery, uniportal vats, vv-ecmo

## Abstract

Pneumothorax after a pneumonectomy is a rare but potentially fatal condition due to markedly reduced pulmonary reserve. Preventing recurrence is crucial in this population. When surgery is required, intraoperative ventilation is particularly challenging. Options include conventional strategies such as high-frequency jet ventilation or endobronchial blockade, or extracorporeal membrane oxygenation (ECMO) when apnea tolerance is limited. We report the case of a 79-year-old male with a history of a left pneumonectomy for lung cancer, chronic obstructive pulmonary disease, and pulmonary emphysema who presented with sudden-onset, right-sided chest pain. Chest radiography revealed a right-sided pneumothorax, which was managed with chest tube drainage. A chest CT demonstrated a large bulla in the middle lobe. The pneumothorax recurred a few days later, requiring the reinsertion of an 18-Fr chest drain. The patient was evaluated by the thoracic surgery team but was deemed high risk, and chemical pleurodesis with talc slurry was performed through the chest drain to prevent recurrence. Two days after discharge, the pneumothorax recurred. Following recurrent episodes despite drainage and talc pleurodesis, the case was discussed in a multidisciplinary team involving pulmonology, thoracic surgery, and intensive care. Given his age, frailty, comorbidities, and severely reduced pulmonary reserve, conventional ventilation strategies were considered unsafe, and ECMO was selected to permit definitive surgical treatment. He underwent venovenous femoro-jugular ECMO cannulation, followed by uniportal video-assisted thoracic surgery with wedge resection of the middle-lobe bulla and combined chemical pleurodesis with talc and mechanical pleurodesis by pleural abrasion, without complications. He was extubated on the day of surgery and decannulated the following day. Postoperative Doppler ultrasonography revealed deep venous thrombosis of both cannulated vessels, and anticoagulation with low-molecular-weight heparin was initiated. The patient was discharged on postoperative day 12, with a switch to the direct oral anticoagulant edoxaban. No recurrence was observed at the five-month follow-up. Recurrent pneumothorax in a post-pneumonectomy patient represents a high-risk scenario in which conventional ventilation may not safely permit surgery. ECMO-assisted surgery offers a viable option for definitive management in patients with prohibitive pulmonary function.

## Introduction

After a pneumonectomy, profound compensatory changes occur, leading to a progressive decline in pulmonary function and markedly reduced pulmonary reserve. Therefore, any additional intervention on the single remaining lung represents a significant challenge [1,2]. Pneumothorax in a post-pneumonectomy patient, although rare, is a potentially life-threatening condition, and preventing recurrence is particularly important given the risk of rapid decompensation [3]. When surgical intervention is required to prevent recurrence, achieving safe intraoperative ventilation becomes especially challenging. Conventional strategies, such as high-frequency jet ventilation [4] or selective lobar or segmental blockade using an endobronchial blocker [5-7], may be feasible, but these patients often have very limited tolerance for apnea and a high risk of hypercapnia, making adequate surgical exposure difficult to achieve. In such scenarios, extracorporeal membrane oxygenation (ECMO) provides an alternative means of ensuring oxygenation and ventilation while allowing complete lung collapse and optimal operative conditions [8,9].

We report the case of a recurrent pneumothorax in a post-pneumonectomy patient in whom conservative management failed to prevent recurrence, necessitating definitive surgical treatment. ECMO was used to safely support ventilation during surgery, enabling both adequate exposure and successful management of this complex and high-risk condition.

## Case presentation

We report the case of a 79-year-old male with an Eastern Cooperative Oncology Group performance status of 1. He was a former smoker with a 50-pack-year history and had a past medical history of chronic obstructive pulmonary disease, pulmonary emphysema, and left nephrectomy for kidney adenocarcinoma. In 2018, he underwent a left pneumonectomy with lymphadenectomy for lung cancer (pathological stage pT1bN0M0) and subsequently completed five years of surveillance without evidence of disease recurrence.

**Figure 1:**
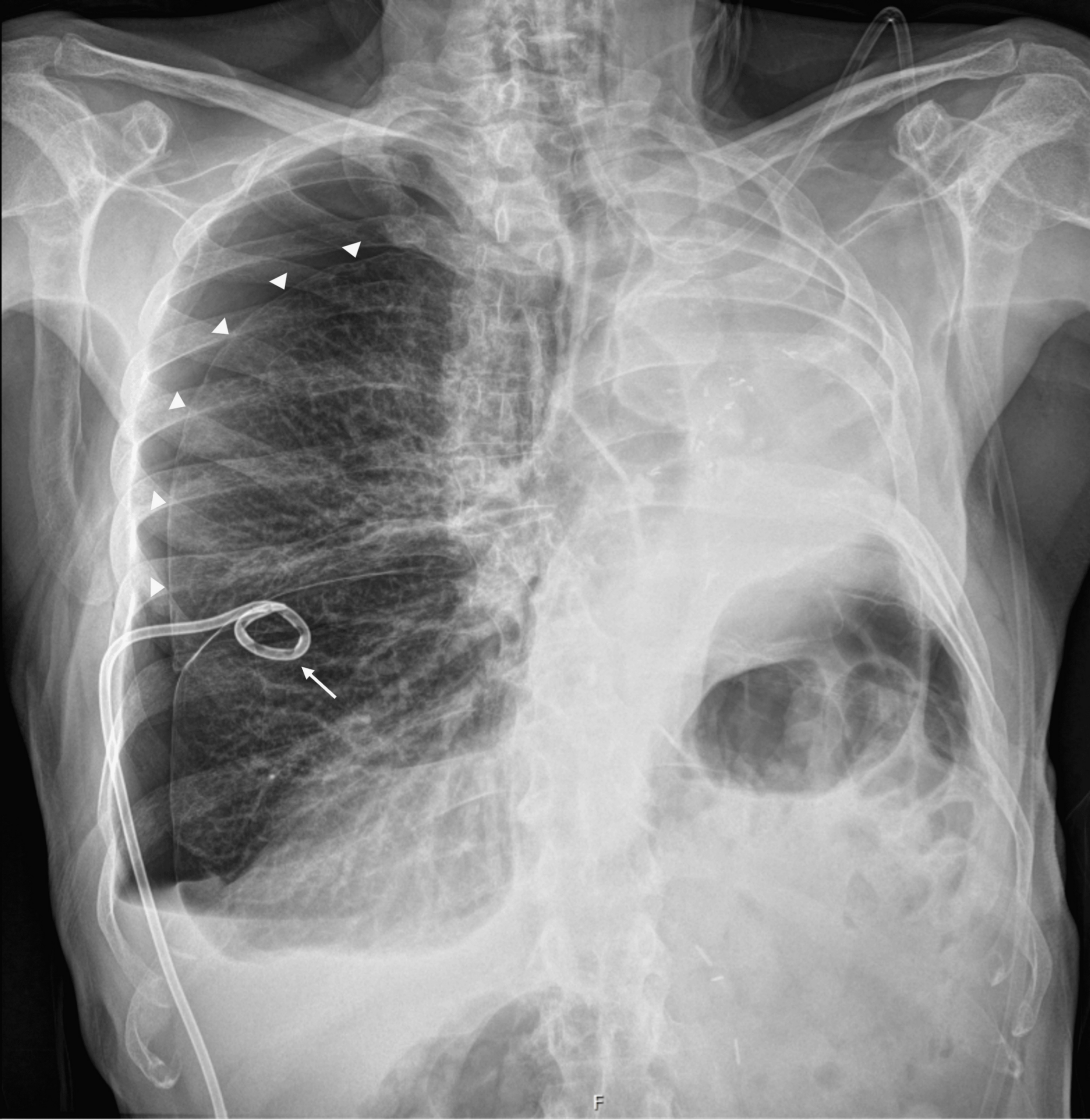
Chest radiography after chest tube insertion. Post-procedure chest radiograph demonstrating partial re-expansion of the right lung following the insertion of a 14-Fr pigtail chest tube. Arrowheads indicate the visceral pleural line, consistent with a residual pneumothorax, and the arrow denotes the pleural drainage catheter. The changes observed on the left side are consistent with a prior pneumonectomy.

He presented to the emergency department with sudden-onset right anterior chest pain, worsened by inspiration. On arrival, he was polypneic (30 breaths per minute), with a peripheral oxygen saturation of 96% on room air, and was normocardic and normotensive. Pulmonary auscultation revealed decreased breath sounds on the right. Arterial blood gas analysis showed no evidence of respiratory failure, myocardial injury biomarkers and D-dimer levels were normal, and the ECG revealed no acute ischemia. Chest radiography demonstrated a right-sided total pneumothorax (imaging from the initial presentation could not be retrieved due to prior assessment at a private hospital). A 14-Fr pigtail chest tube was inserted, resulting in clinical improvement; however, post-procedure imaging demonstrated only partial lung re-expansion (Figure 1).

**Figure 2:**
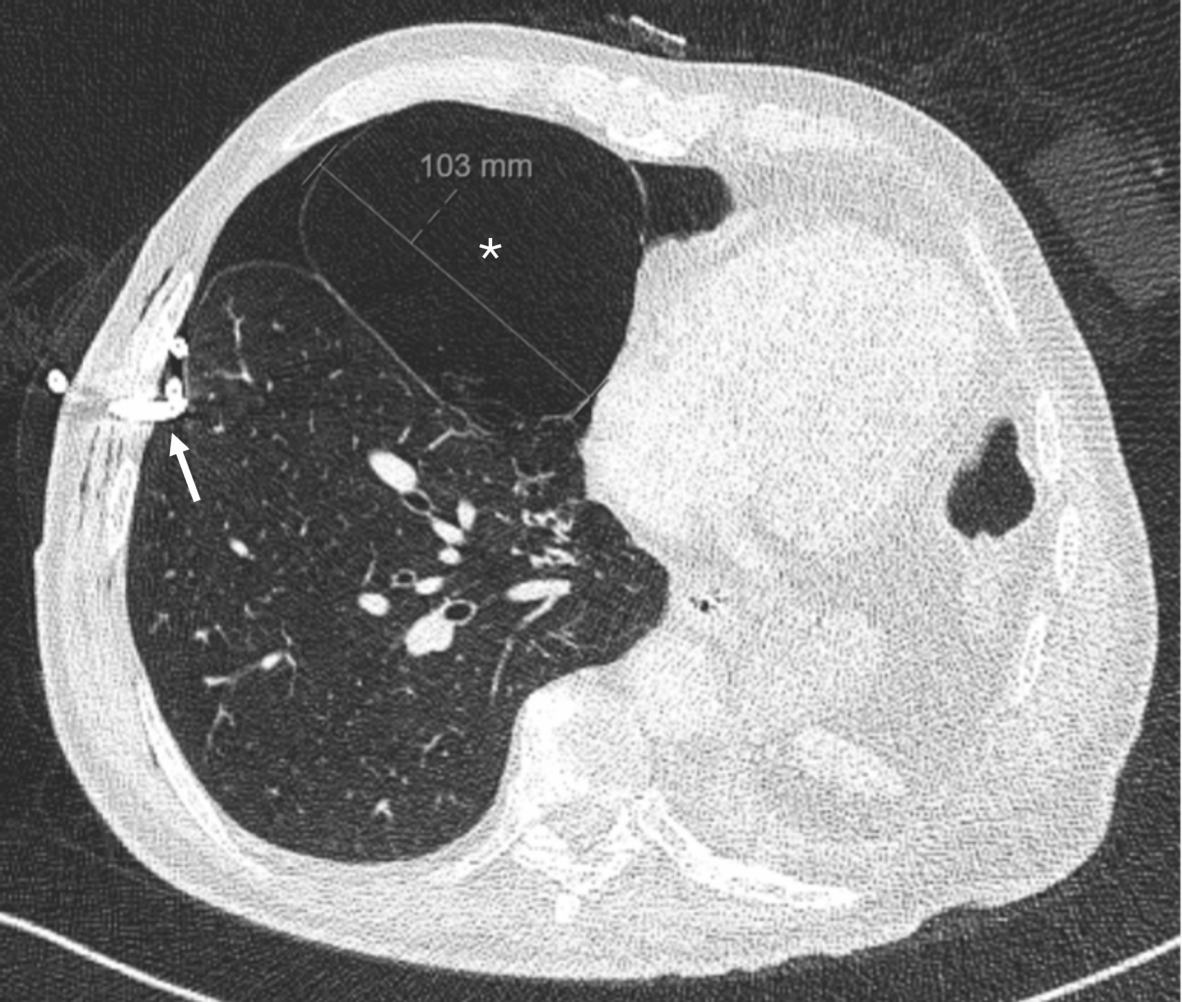
Chest CT scan. Axial chest CT scan demonstrating a large emphysematous bulla in the right middle lobe (asterisk), measuring approximately 103 mm in its greatest diameter. The arrow indicates the pigtail chest tube in situ.

By hospital day four, the air leak persisted, prompting a chest CT scan that demonstrated leftward mediastinal deviation and left pleural effusion related to the prior pneumonectomy, centrilobular and paraseptal emphysema, and a large emphysematous bulla in the middle lobe measuring 103 mm in its greatest dimension (Figure 2).

**Figure 3:**
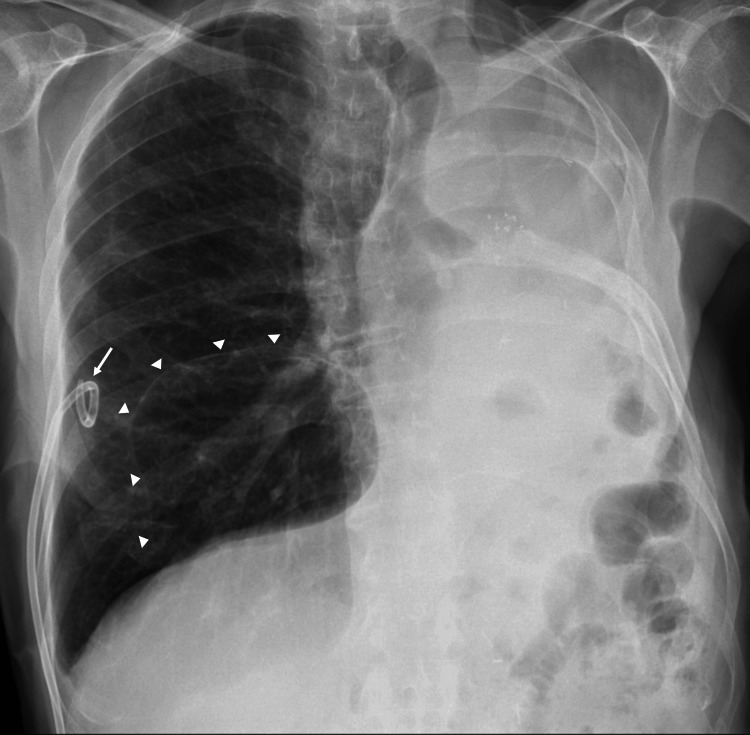
Chest radiograph demonstrating complete re-expansion of the right lung. Chest radiograph demonstrating full re-expansion of the right lung following resolution of the air leak. Arrowheads indicate the middle-lobe bulla seen on the CT scan, and the arrow denotes the pigtail pleural drainage catheter.

On hospital day six, thoracic surgery was consulted; however, due to the patient’s age and comorbidities, surgical risk was deemed too high. Conservative management with ongoing pleural drainage was continued. After 12 days, the air leak resolved, and chest radiography confirmed full right lung re-expansion (Figure 3). The chest tube was removed, and the patient was discharged.

**Figure 4:**
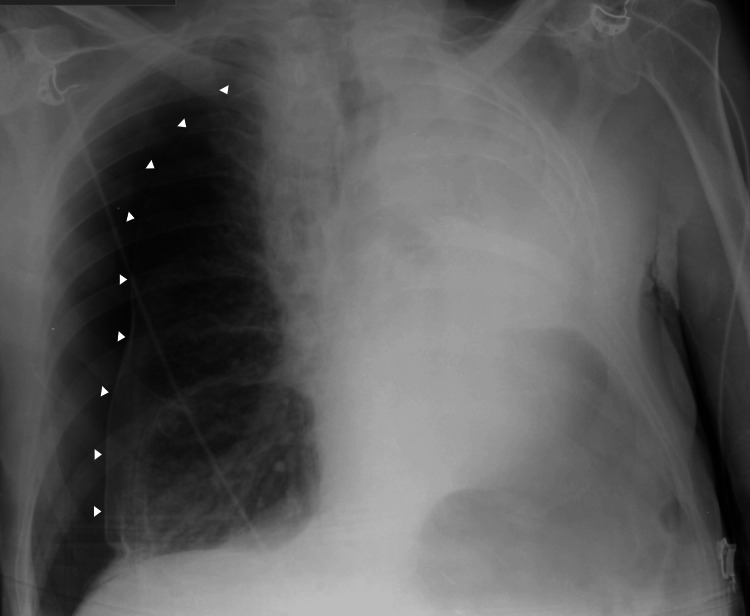
Recurrent right-sided pneumothorax. Chest radiograph obtained at readmission demonstrating a recurrent right-sided pneumothorax. Arrowheads delineate the visceral pleural line, consistent with lung collapse.

Four days later, he returned with sudden-onset dyspnea and was again polypneic but maintained normal peripheral oxygen saturation on room air. Chest radiography revealed recurrent right-sided pneumothorax (Figure 4), and an 18-Fr chest tube was placed, with a residual pneumothorax persisting afterward. Arterial blood gas analysis continued to show no evidence of respiratory failure, and biomarkers and ECG remained unremarkable.

**Figure 5:**
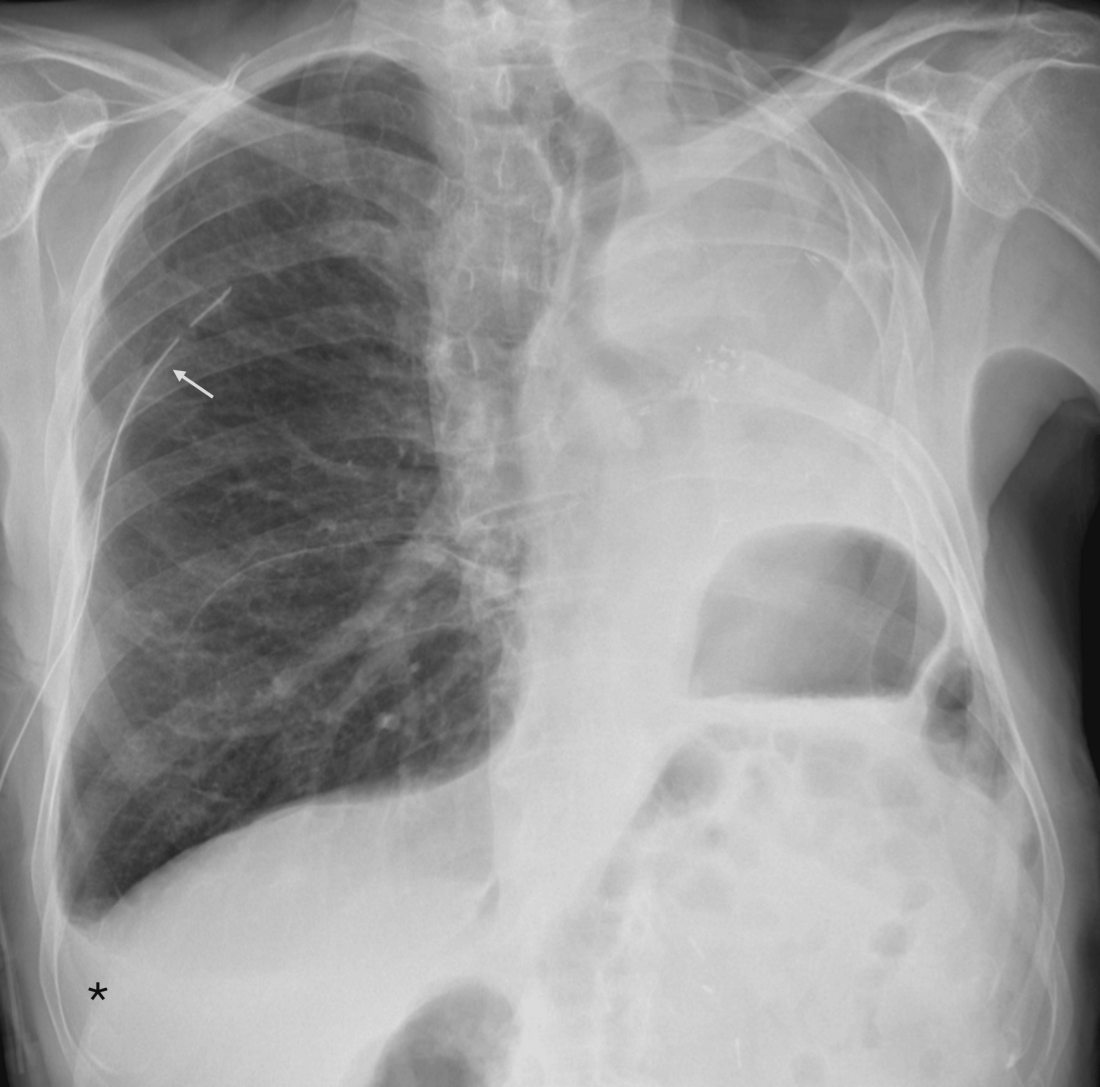
Post-pleurodesis chest radiography. Chest radiograph after talc pleurodesis showing full lung expansion. The asterisk indicates a small residual pleural effusion. The arrow denotes the chest tube in place.

Seven days into his hospital stay, given the recurrent pneumothorax and persistent air leak, the case was re-evaluated by the thoracic surgery team. However, the patient remained a high surgical risk. To prevent recurrence, chemical pleurodesis with talc slurry was performed through the chest tube without complications. Post-procedure imaging confirmed full lung expansion with a small residual pleural effusion (Figure 5). Two days later, the air leak resolved, and the patient was discharged home.

**Figure 6:**
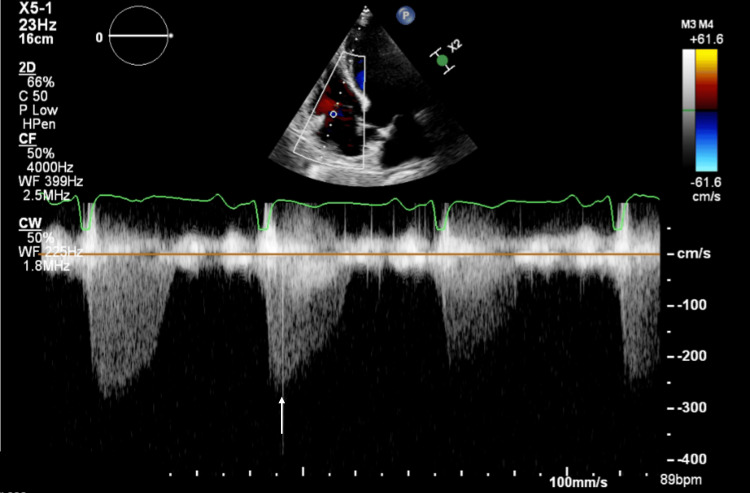
Transthoracic echocardiography. Transthoracic echocardiography showing a systolic tricuspid regurgitation jet on continuous-wave Doppler, representing systolic flow from the right ventricle to the right atrium. The arrow indicates the peak systolic velocity of the tricuspid regurgitation jet, reflecting the right ventricle–right atrium pressure gradient used for indirect estimation of pulmonary artery systolic pressure.

Two days after discharge, the patient returned with sudden-onset dyspnea, and chest radiography once again demonstrated a right-sided pneumothorax. An 18-Fr chest tube was inserted, resulting in full lung re-expansion. After 12 days of persistent air leak, a multidisciplinary team (MDT) involving pulmonology, thoracic surgery, and intensive care convened to determine the best approach to prevent further pneumothorax recurrence. Pulmonary function tests demonstrated a severe obstructive component, with a forced expiratory volume in the first second (FEV₁) of 40% of predicted, and a diffusion impairment with a diffusing capacity of the lung for carbon monoxide (DLCO) of 61%, as summarized in Table 1. Given the patient’s frailty, his single-lung physiology with severely reduced pulmonary reserve, and the predictable low tolerance for apnea during surgery, the MDT considered ECMO the safest strategy to enable surgical management. A preoperative evaluation was performed to ensure safe ECMO cannulation. The patient’s blood workup demonstrated no significant abnormalities and is detailed in Table 1. Transthoracic echocardiography demonstrated preserved biventricular systolic function, an estimated pulmonary artery systolic pressure of 29 ± 5 mmHg, without evidence of pulmonary hypertension, and no significant age-related valvular disease (Figure 6). Doppler ultrasonography confirmed patency of the jugular veins and femoral arteries and veins, with normal flow patterns (images were not available as the examination was performed at the patient’s bedside).

**Table 1:** Preoperative laboratory and pulmonary function test results. FEV₁: forced expiratory volume in the first second; DLCO: diffusing capacity of the lung for carbon monoxide

		Results	Reference values
Preoperative blood workup	Hemoglobin	14.6 g/dL	13–18 g/dL
	Hematocrit	38.9%	43–55%
	White blood cell count	6.95 x 10^9^/L	4–11 x 10^9^/L
	Platelets	161 x 10^9^/L	150–400 x 10^9^/L
	Creatinine	1.07 mg/dL	0.67–1.17 mg/dL
	C-reactive protein	7.8 mg/L	<3 mg/L
Pulmonary function tests	FEV₁	40%	% of predicted
	DLCO	61%	% of predicted

By day 18 of his hospital course, the patient was electively admitted to the intensive care unit, where he was sedated, intubated with a single-lumen tube, and underwent venovenous femoro-jugular ECMO cannulation without complications. Surgery was performed the same day. A right uniportal video-assisted thoracic surgery (VATS) was performed. Before lung deflation, the patient was ventilated with a positive end-expiratory pressure (PEEP) of 5 cmH₂O. To achieve optimal surgical exposure, the endotracheal tube was then disconnected from the ventilator, allowing complete lung collapse. Intraoperative findings included mild apical adhesions (left untouched to avoid additional air leak) and a large bulla in the middle lobe. A wedge resection of the middle lobe containing the bulla was performed using a stapling device. The lung was then reinflated, and a saline leak test showed no evidence of air leak. Pleurodesis by pleural abrasion and talc insufflation was performed, and a single apically orientated chest tube was placed. No immediate postoperative air leak was observed through the ventilator or chest tube. Throughout the procedure, the patient remained hemodynamically stable, with peripheral oxygen saturation between 98% and 100%, normal heart rate and blood pressure, and a partial pressure of arterial carbon dioxide (PaCO₂) of 33-35 mmHg.

**Figure 7:**
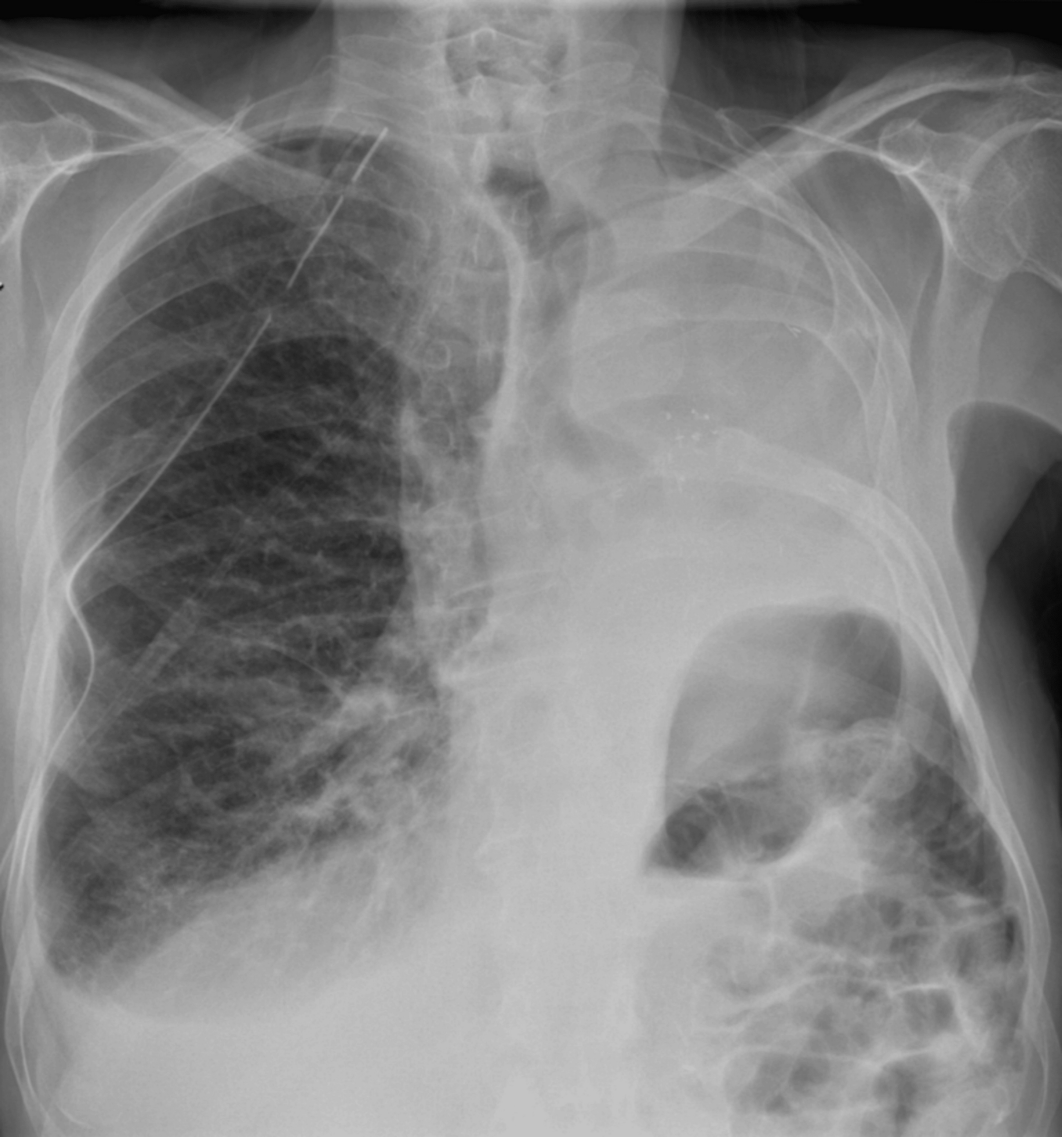
Postoperative chest radiograph. Postoperative chest radiograph demonstrating full expansion of the right lung following surgery, with no evidence of residual pneumothorax and a small residual pleural effusion.

Postoperative chest radiography showed a fully expanded right lung (Figure 7). The patient was extubated on the same day in the intensive care unit, without signs of respiratory distress. On postoperative day one, no air leak was observed, the lung remained expanded, and ECMO was successfully decannulated. Doppler ultrasonography performed after decannulation revealed deep venous thrombosis of both cannulated vessels, and anticoagulation with low-molecular-weight heparin was initiated. The patient was transferred to the ward on postoperative day five, and the chest tube was removed the following day, with no air leak observed after surgery.

**Figure 8:**
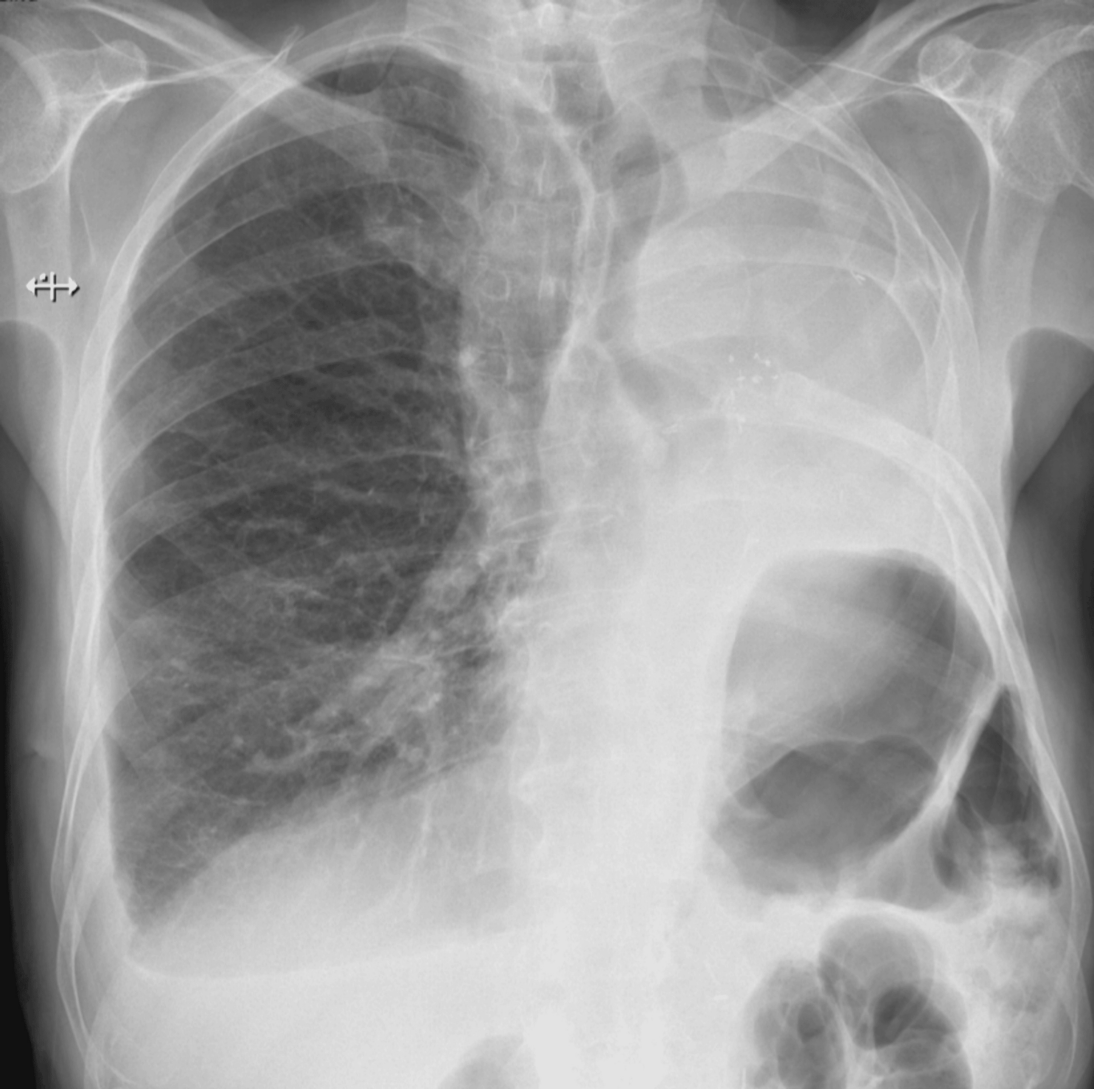
Chest radiograph one month after surgery. Chest radiograph obtained one month after surgery showing complete re-expansion of the right lung and a small residual postoperative pleural effusion, with no evidence of pneumothorax recurrence.

Due to complaints of fatigue on minimal exertion, a transthoracic echocardiogram was performed, which was similar to the preoperative study, showing preserved biventricular systolic function and no other significant abnormalities. After undergoing inpatient respiratory rehabilitation, he was discharged on postoperative day 12 with the direct oral anticoagulant (DOAC) edoxaban. One month after surgery, he reported functional improvement with continued respiratory rehabilitation and remained free of pneumothorax recurrence. Chest radiography showed a fully expanded right lung with a residual postoperative pleural effusion (Figure 8). At the five-month follow-up, no recurrence was observed. The current plan is to continue DOAC therapy for a total of six months.

## Discussion

After a pneumonectomy, significant compensatory changes occur, including ipsilateral mediastinal shift and diaphragmatic elevation, hyperinflation of the remaining lung, and a progressive decline in pulmonary function. The loss of pulmonary vascular bed following surgery leads to increased pulmonary artery pressure and right ventricular workload, predisposing the patient to pulmonary hypertension and right heart failure. Because of these alterations, any additional surgical procedure involving the remaining lung poses a considerable challenge due to diminished cardiopulmonary reserve and high risk of respiratory insufficiency [1,2].

During thoracic surgery, particularly thoracoscopic procedures, an atelectatic lung is essential to achieve adequate surgical exposure. In post-pneumonectomy patients, obtaining satisfactory exposure of the remaining lung is particularly demanding. In addition, these patients are at increased risk of barotrauma associated with mechanical ventilation. Therefore, one-lung-protective ventilation principles should be followed. These include the use of a low tidal volume (TV) of 6 mL/kg or less of ideal body weight and a PEEP of at least 5 cmH₂O (as low TV combined with low PEEP increases the risk of atelectasis) [10]. Various ventilation strategies have been described for such cases, including intermittent apnea [11], high-frequency jet ventilation [4], and selective lobar or segmental blockade using an endobronchial blocker [5-7] or a double-lumen endotracheal tube [12]. To minimize barotrauma, surgery under spontaneous breathing with local and epidural anesthesia has also been reported [13].

However, long periods of apnea required to achieve lung deflation during surgery may not be feasible in these patients. Selective lobar or segmental ventilation may also be insufficient to provide adequate surgical exposure, particularly in emphysematous patients. These difficulties are further exacerbated during video-assisted procedures. In addition, such ventilation strategies place these patients at risk of intraoperative hypercapnia, which can delay postoperative weaning from mechanical ventilation and increase the likelihood of further complications. ECMO offers an alternative means to maintain adequate oxygenation and normocapnia while providing optimal surgical exposure. It can also facilitate immediate postoperative extubation and early decannulation. However, the risks of cannulation-site thrombosis and intraoperative bleeding due to systemic heparinization must be carefully weighed [4,8]. There are reports in the literature describing the successful use of ECMO in the surgical management of contralateral pneumothorax after pneumonectomy (CPAP) [8,9]. CPAP is a rare but potentially fatal condition, with an incidence ranging from 0.3% to 1.2% and a mortality rate approaching 50% [3].

If a CPAP is suspected or confirmed, immediate drainage is recommended, even before radiological confirmation. In cases of persistent air leak, talc administration through the chest tube (talc slurry) has been reported with successful resolution [14]. Because recurrence is a relevant concern in these patients, definitive treatment should be pursued whenever feasible. When surgery is considered, the choice of technique must be guided by the patient’s residual pulmonary function; predicted FEV₁ and DLCO values above 40% of the predicted indicate an acceptable operative risk. In addition, preoperative cardiac ultrasound is essential to exclude pulmonary hypertension or right heart failure, which constitute contraindications to further lung resection [2]. For patients with prohibitive surgical risk, pleurodesis with talc slurry remains a reasonable alternative to reduce the likelihood of recurrence [15].

The management of recurrent CPAP is challenging, and the approach to these patients should be multidisciplinary and individualized. This patient’s advanced age, single-lung physiology with markedly reduced pulmonary reserve, and predictable low tolerance for apnea made conventional ventilation strategies unsafe, increasing the risk of hypercapnia, postoperative complications, and poor surgical exposure. In this context, ECMO provided a safe and effective means of maintaining adequate oxygenation and ventilation while allowing optimal surgical conditions, thereby enabling definitive treatment with minimized perioperative risk. Given the rarity of CPAP and its potentially fatal consequences, the successful use of ECMO-supported surgery in our patient adds valuable insight into the management of similarly high-risk cases. It further supports the integration of venovenous ECMO into multimodal approaches to thoracic anesthesia and surgery, particularly in patients with severe pulmonary impairment and significant comorbidities undergoing complex thoracic procedures, with the potential to facilitate surgery and enhance postoperative recovery [16].

## Conclusions

Recurrent pneumothorax in a post-pneumonectomy patient is a rare and high-risk condition in which conventional ventilation strategies may be unsafe or insufficient. This case illustrates that ECMO can provide a safe means of maintaining gas exchange while enabling definitive surgical treatment in selected patients with severely limited pulmonary reserve. ECMO-assisted surgery should be considered when recurrence prevention is required, and operative risk is otherwise prohibitive.
